# Factors associated with antenatal care adequacy in rural and urban contexts-results from two health and demographic surveillance sites in Vietnam

**DOI:** 10.1186/1472-6963-12-40

**Published:** 2012-02-15

**Authors:** Toan K Tran, Karin Gottvall, Hinh D Nguyen, Henry Ascher, Max Petzold

**Affiliations:** 1 Family Medicine Department, Hanoi Medical University (HMU), No.1 Ton That Tung Street, Hanoi, Vietnam; 2 Division of Global Health (IHCAR), Department of Public Health Sciences, Karolinska Institute (KI), 171 77 Stockholm, Sweden; 3 Obstetrics and Gynaecology Department, Hanoi Medical University (HMU), No.1 Ton That Tung Street, Hanoi, Vietnam; 4 The Nordic School of Public Health (NHV), Nya Varvet Byggnad 25, Box 12133, SE-402 42 Gothenburg, Sweden; 5 Sahlgrenska Academy, University of Gothenburg (GU), PO Box 400, SE-405 30 Gothenburg, Sweden

**Keywords:** Antenatal care, Socio-economic determinants, Adequacy, Urban and rural, Vietnam

## Abstract

**Background:**

Antenatal Care (ANC) is universally considered important for women and children. This study aims to identify factors, demographic, social and economic, possibly associated with three ANC indicators: number of visits, timing of visits and content of services. The aim is also to compare the patterns of association of such factors between one rural and one urban context in northern Vietnam.

**Methods:**

Totally 2,132 pregnant women were followed from identification of pregnancy until birth in two Health and Demographic Surveillance Sites (HDSS). Information was obtained through quarterly face to face interviews.

**Results:**

Living in the rural area was significantly associated with lower adequate use of ANC compared to living in the urban area, both regarding quantity (number and timing of visits) and content. Low education, living in poor households and exclusively using private sector ANC in both sites and self employment, becoming pregnant before 25 years of age and living in poor communities in the rural area turned out to increase the risk for overall inadequate ANC. High risk pregnancy could not be demonstrated to be associated with ANC adequacy in either site. The medical content of services offered was often inadequate, in relation to the national recommendations, especially in the private sector.

**Conclusion:**

Low education, low economic status, exclusive use of private ANC and living in rural areas were main factors associated with risk for overall inadequate ANC use as related to the national recommendations. Therefore, interventions focussing on poor and less educated women, especially in rural areas should be prioritized. They should focus the importance of early attendance of ANC and sufficient use of core services. Financial support for poor and near poor women should be considered. Providers of ANC should be educated and otherwise influenced to provide sufficient core services. Adherence to ANC content guidelines must be improved through enhanced supervision, particularly in the private sector.

## Background

Nearly 4 million neonatal deaths and 500,000 maternal deaths are estimated to occur annually in the world. About 98% of these occur in low and middle income countries [1]. Ante-Natal Care (ANC) has been proven to be effective in improving pregnancy outcomes through early detection and management of pregnancy complications [2]. Lack of relevant and high quality antenatal care is still a major concern for many pregnant women in low and middle income countries in Africa and Asia where the number of women who seek routine antenatal care is low and they often do so only late in pregnancy [2,3].

Many studies have been conducted to examine factors related to the utilization of ANC in middle and low income countries [4-6]. Maternal education, household income, parity, women's age and occupation, cost and availability of services are factors commonly correlated to the use of ANC in these studies [7]. The place of residence, urban or rural area, is also a factor affecting the use of ANC. Some studies have shown differences between the urban and rural areas in knowledge, attitude and practices of women towards antenatal care [8,9].

WHO recommends four ANC visits at 4^th^, 6^th ^or 7^th^, 8^th ^and 9^th ^month for women with normal pregnancy in middle and low income countries. National ANC recommendations vary between countries regarding number of visits, timing of visits and service contents of visits. In Vietnam, the national recommendations for uncomplicated pregnancies are at least three ANC visits, one in each trimester with adequate services during each visit. In this paper we use the word adequacy to describe the minimal required ANC as defined by the Vietnamese recommendations and denote these: adequate time for first visit, adequate number of visits and adequate service content. When all are satisfied we shall talk about overall adequate ANC.

The recommended ANC package of services includes measurements of body weight and height, blood pressure, symphysis-fundus measurement, abdominal circumference, fetal heart rate; vaginal examination; urine protein test; blood tests for anemia and HIV; tetanus vaccination; folate combined iron supplement; malaria chemoprophylaxis and prenatal medical consultation [10]. Ultrasound examination is not officially recommended for pregnant women but is available in all hospitals and most private clinics.

In 2002, 20% of all pregnant women still did not use any ANC visit during their pregnancy and only 54.3% used three or more visits (72.6% in urban and 48.4% in rural areas) [11]. The service content of the ANC was neither addressed in the national health survey [11] nor in the national health statistics profile [12].

Some cross sectional studies of ANC determinants have been conducted in Vietnam, mostly in rural areas with a focus primarily on individual maternal factors. Mother's education, occupation and number of children were found to be important factors for explanation of ANC use variation [13-16]. The national health survey in 2002 showed that living in urban areas appears to affect the use of ANC [11]. There is no study on the underlying factors associated with variations in ANC utilization in urban settings.

The aim of this study is to identify factors, demographic, social and economic, possibly associated with three ANC adequacy indicators: number of visits, timing of visits and content of services. The aim is also to compare the patterns of associations between ANC use and these factors between one rural and one urban context in northern Vietnam.

## Methods

### Study setting

The study was carried out in two health and demographic surveillance sites (HDSS): FilaBavi in Bavi rural district and DodaLab in Dongda urban district in Hanoi, Vietnam. FilaBavi was established in 1999, covering all households in 69 randomly selected clusters from totally 352 in Bavi district. The population under surveillance comprises about 11,000 households with 51,000 persons that is, about 20% of the entire district population [17]. DodaLab was established in 2007 with three out of 21 communes, strategically selected at different socioeconomic levels. All households in these three communes together including about 11,000 households with 38,000 persons and being 12% of the total district population, are under surveillance.

In DodaLab 78.6% of all adults have graduated from at least high school. This can be compared to 31.5% in FilaBavi. More than 50% of the adults in FilaBavi are farmers while the most common occupation in DodaLab is office employment (33.3%) and business activities (20.1%). The median of reported annual income in DodaLab in 2009 is about 1,200 USD per capita, almost three times that in FilaBavi [18]. Core ANC services are available at almost every commune health centre (CHC) in both sites.

Several public hospitals at different levels and many private clinics are situated in Dong Da. Bavi has only one district hospital and fewer private clinics. The average road distances to access the nearest public hospital, estimated by respondents, are 1.8 km in DodaLab and 10.2 km in FilaBavi. In both sites, women can receive ANC either in public or in private health facility.

In the two HDSSs, 106 field workers, mostly female have been recruited and trained. They are responsible for collecting data through household interviews using structured questionnaires. The routine data collection comprises a household survey repeated every two years to collect and update demographic and socioeconomic information at individual and household level together with quarterly follow up surveys to record individual health and demographic events [18].

### Study design

The study was conducted using two cohorts of pregnant women, recruited in parallel in DodaLab and FilaBavi HDSS from April 2008 to December 2009. During this period, 2,757 pregnant women were identified through the routine surveys in the two sites. These women were then followed quarterly until termination of the pregnancy. Altogether, 625 women who missed two consecutive interviews due to out-migration (94); late in-migration or late identification of pregnancy (383); miscarriage or induced abortion (148) were excluded. In total, 2,132 women were followed until birth and included in the analysis of the study (814 in DodaLab and 1,318 in FilaBavi).

### Data collection

Information about the use of antenatal care was obtained through household interviews every three months using a structured questionnaire specific for this study together with the routine HDSS instruments. The women were interviewed about the number of ANC visits, time for the first visit, service content of ANC visits and cost for ANC utilization.

Data was collected by the HDSS interviewers, who were trained carefully in household interviewing and the content of the questionnaire. For data quality control, a random sample of three percent of the pregnant women was re-interviewed by field supervisors. There was a good correspondence between interview and re-interview showing 12% in DodaLab and 9% in FilaBavi with some mismatch, mostly on date of the last menstruation and date of interview. All forms were rechecked by field supervisors and data clerks before entering into databases. Demographic and socioeconomic information was taken from the latest household survey in the two HDSSs conducted in 2009.

### Explanatory variables, factors possibly associated with the use of ANC

To study relations and to explain variation in ANC adequacy indicators we use a number of explanatory variables. For the individual woman we consider age, education, occupation, marital status, parity and high risk that is, the women meets at least one of the following criteria: *nullipara *and aged 40 years or more, multipara with four or more childbirths, women reporting any of the following complications during previous pregnancies: miscarriage, preterm delivery, caesarean section, stillbirth, or neonatal death or any of the following conditions: high blood pressure, diabetes, epilepsy or depression, during the current pregnancy [19].

Household economic status was measured using a wealth index estimated by Principal Component Analysis of all household variables describing housing and assets. The wealth index scores were grouped into terciles, each containing one third of all households: poor (1^st ^tercile); middle (2^nd ^tercile) and rich (3^rd ^tercile). Women were classified according to the tercile of their households. The community socioeconomic condition was classified in different ways in the two areas. In DodaLab, the three communes have been strategically selected with different socioeconomic levels according to the official classification by local authorities. In FilaBavi there are three types of geographical area: mountainous, highland and lowland. These differ in reported income per capita and mean distances to the nearest health facility. In the economic analysis they were considered as low, middle and high level, respectively. It is important to note that the concepts of community socioeconomy are not comparable between the areas.

### Antenatal care variables, outcome variables

In this study, an ANC visit was defined as a visit to a health worker for checking and supervision of pregnancy without illness being the reason. Adequate use of ANC or ANC adequacy was defined using three basic indicators: ***early attendance ***(first ANC visit made during first trimester); ***enough visits ***(at least three ANC visits) and ***sufficient services ***(all core services performed at least once during the pregnancy). Overall adequate use of ANC was defined as all these three indicators being satisfied. A woman does not have overall adequate use if one or more of the basic indicators is not satisfied.

According to the national Vietnamese guidelines, ANC services that is the actual actions taken during a visit, can be classified as follows:

**• *Core services ***are recommended for all pregnancies and include assessments of body weight and height, blood pressure, fetal examination (symphysis-fundus, abdominal circumference, fetal heart rate); urine test; tetanus vaccination and prenatal medical consultation.

**• *Optional services ***are not recommended for all pregnant women either since they are just applicable for specific groups (folate combined iron supplement for women in areas with high prevalence of anemia; malaria chemoprophylaxis for women in epidemic areas; vaginal examination for women with medical indications) or they are not available at most commune health centers, where women with a normal pregnancy are recommended to have ANC. Women reporting about their ANC experiences do not know exactly what their blood test are taken for so we cannot discuss how often test for anemia and HIV are taken [10].

Ultrasound use is not recommended but was also asked during the interviews.

### Data analysis

Data was entered into computers with Access software and analyzed using STATA software version 11.0. Description and analysis were conducted separately for the urban and the rural area. Both simple and multiple regression models were used to study the statistical associations between the explanatory and the ANC outcome variables. The bivariate analysis shows associations as they appear in the context of all other variables and their variations and covariation. The multivariate analysis aims to study the associations between one dependent and one independent variable under the condition that all other variables are fixed.

Some variables for example education, occupation and economic status might be strongly correlated and technically influence the results of regression analyses that include all available variables. However, in our study, the results were not much different when we run the models e.g. with and without the occupation variable. There is a correlation between education and occupation but not very strong. Another example is age and parity. There is a correlation between the two variables but not strong enough to cause problems if both variables are included.

### Ethical considerations

The two HDSS were developed in collaboration with the local authorities. They were also approved by the Scientific and Ethical Committee of Hanoi Medical University and the Ministry of Health of Vietnam. The participants were informed about the purpose of the project, their rights to decline participation and to withdraw at any stage of the study. Verbal consent was obtained from all pregnant women. Pregnant women could receive advice from obstetric experts within the project for problems they may have during pregnancy or in the use of antenatal care. A small gift worth about 2 USD was offered to each newborn baby in the first interview after delivery.

## Results

### Characteristics of the pregnant women

Table 1 shows that around half of the women were nullipara (46.6% in the rural and 56.5% in the urban area) or expected a second child (35.4% in the rural areas and 39.1% in the urban). The mean ages for nulliparas were 23.5 and 26.7 years, respectively for the rural and urban areas. As much as 57% of the rural women had graduated from secondary school or less while 62.3% of the urban women had post-high school education. Self employment accounted for 36.1% in the urban and 79.7% in the rural areas. *Kinh *is the majority ethnic group in both sites. Women in other groups accounted for 0.7% in the urban area and 5.5% in the rural. Thirteen percent% of the rural women and 11% of the urban women were classified as high risk pregnancy. The rural women used more private and less public ANC services than those in the urban area.

**Table 1:** Percentages of women reporting positive with respect to the three basic indicators of ANC utilization and care, by demographic, social and economic characteristics

	Rural areas				Urban areas			
	**All women**	**Adequate visits (%)**	**Early use (%)**	**Sufficient services (%)**	**All women**	**Adequate visits (%)**	**Early use (%)**	**Sufficient services (%)**

***Age group***				**				
< 25	604	*77.3*	*67.7*	*16.2*	109	*95.4*	*99.1*	*81.7*
25-34	625	*77.4*	*70.6*	*24.0*	626	*97.6*	*97.0*	*81.5*
35+	89	*74.2*	*68.5*	*22.5*	79	*96.2*	*96.2*	*77.2*
***Education***		***	***	***		***	*	
Secondary or less	748	*71.9*	*64.0*	*16.4*	48	*87.5*	*91.7*	*72.9*
High school	350	*78.9*	*69.7*	*19.1*	259	*97.7*	*96.5*	*81.8*
Post high school	220	*92.3*	*85.5*	*35.5*	507	*97.8*	*98.0*	*81.5*
***Occupation***		***	***	***		**		
Employment	267	*88.8*	*84.6*	*33.0*	520	*98.5*	*97.3*	*82.5*
Self employment	1051	*74.2*	*65.2*	*17.1*	294	*94.9*	*96.9*	*78.6*
***Ethnic group***		***				*	*	
Kinh	1245	*78.2*	*68.7*	*20.7*	808	*97.3*	*97.3*	*81.3*
Minority	73	*60.7*	*76.7*	*13.7*	6	*83.3*	*83.3*	*50.0*
***Household economic***		***	***	***				
Low (1^st ^tercile)	412	*68.9*	*63.8*	*14.3*	235	*95.7*	*96.6*	*77.0*
Middle(2^nd ^tercile)	428	*76.7*	*67.1*	*20.1*	292	*96.6*	*96.6*	*82.5*
Rich (3^rd ^tercile)	458	*84.5*	*75.6*	*26.3*	287	*99.0*	*98.3*	*82.9*
***Community condition***		***		***				**
Low	232	*72.4*	*73.3*	*14.7*	354	*96.3*	*96.6*	*76.6*
Middle	780	*75.1*	*66.5*	*18.8*	213	*97.2*	*98.1*	*88.7*
High	306	*86.0*	*72.6*	*28.8*	247	*98.4*	*97.2*	*81.0*

***Total***	***1318***	***77.2***	***69.1***	***20.3***	***814***	***97.2***	***97.2***	***81.1***

*p *value for Chi square test of the null hypothesis that all true percentages are equal within a group (no tests for trends). *: *p *< 0.05; **: *p *< 0.01; ***: *p *< 0.001

### Different indicators of adequate use of ANC

Tables 1 and 2 present the percentages of women in each setting who met the criteria for different indicators of adequate ANC utilization. According to Table 1, the urban women received more adequate ANC for all three indicators than those in the rural areas. Only about one fifth of the rural women received sufficient core services according to the national recommendations. The percentages of women who had enough visits, attended ANC early and received sufficient services in the urban areas were 1.3, 1.4 and 4.0 times those in the rural, respectively.

**Table 2:** Percentages of women reporting positive with respect to the three basic indicators of ANC utilization and care, by obstetric and health seeking practice factors

	Rural areas				Urban areas			
	**All women**	**Adequate visits (%)**	**Early use (%)**	**Sufficient services (%)**	**All women (%)**	**Adequate visits (%)**	**Early use (%)**	**Sufficient services (%)**

***Parity***		***	*					
1	614	*82.3*	*72.5*	*22.2*	460	*97.6*	*96.7*	*82.6*
2	466	*71.7*	*65.9*	*18.5*	318	*96.2*	*97.8*	*80.2*
3+	238	*73.9*	*66.8*	*19.3*	36	*100*	*97.2*	*69.4*
***Pregnancy at risk***								
Low risk	1142	*77.0*	*68.9*	*21.0*	722	*97.0*	*97.1*	*81.0*
High risk	176	*78.4*	*70.5*	*15.9*	92	*98.9*	*97.8*	*81.5*
***Type of ANC facilities***		***	***	***		**	**	
Public only	386	*61.7*	*63.7*	*26.7*	459	*96.3*	*96.3*	*81.1*
Private only	116	*62.9*	*63.8*	*5.2*	40	*97.5*	*100*	*62.5*
Both	764	*92.2*	*77.4*	*20.8*	312	*99.0*	*99.0*	*84.3*
***Time for first visit***		***		***		***		*
In1^st ^trimester	1017	*87.4*		*23.3*	791	*97.6*		*81.5*
2^nd^-3^rd ^trimester	301	*54.3*		*13.8*	23	*82.6*		*65.2*

*p *value for Chi square test of the null hypothesis that all true percentages are equal within a group (no tests for trends). *: *p *< 0.05; **: *p *< 0.01; ***: *p *< 0.001

In the rural areas, low education, being self employed or being poor reported less adequate use for all three aspects of ANC utilization. The women living in poor communities were less likely to use adequate numbers of visits and sufficient services. Women aged less than 25 years received significantly less sufficient services (*p *< 0.05) than older women.

In the urban areas, being less educated was significantly associated with late ANC attendance and low use of ANC visits. Receiving fewer visits was more common among self employed women. Women living in poor communities, both sites, received less sufficient services. Women in the minority ethnic group used significantly more ANC visits in the two areas and had earlier ANC attendance in the urban.

In both sites, the percentages of all indicators of ANC use among women with high risk pregnancy were similar to the percentages for those with low risk. Exclusive use of ANC in the private sector was significantly associated with later attendance, insufficient use of core services and non-adequate overall use. The women who attended ANC during the first trimester also used significantly more ANC visits and services. In the rural areas, being a multiparous was associated with later attendance of ANC and fewer ANC visits.

### Factors significantly associated with overall adequate use of ANC

The percentage of overall adequate use of ANC in the urban areas was 5.2 times that in the rural (Table 3). In the two areas, the percentage of overall adequate use of ANC was significantly lower among women with secondary school education or less, poor women and women who exclusively used private ANCs. Self employed women and women living in poor communities were less likely to receive overall adequate ANC in the rural area. Rural women who were less than 25 years old had lower chance to receive overall adequate ANC. Multiparous women seemed to receive lower overall adequate ANC although statistical significance was not observed in either site. High risk pregnant women were not demonstrated to receive more overall adequate use than normal risk women (Table 3).

**Table 3:** Results of simple and multiple logistic regression models with adequate use of ANC as outcome, related to selected explanatory variables in the two areas

	Rural areas			Urban areas		
	**Adequate use (%)**	**Crude OR (95%CI)**	**Adjusted OR (95% CI)**	**Adequate use (%)**	**Crude OR (95%CI)**	**Adjusted OR (95% CI)**

***Age group***	**					
< 25	11.6	0.56 (0.41-0.78)	0.58 (0.40-0.84)	78.0	0.94 (0.58-1.55)	1.06 (0.61-1.84)
25-34	18.9	1	1	78.9	1	1
35+	13.5	0.67 (0.35-1.27)	0.84 (0.41-1.73)	73.4	0.74 (0.43-1.26)	0.86 (0.48-1.54)
***Education***	***			*		
Secondary or less	10.7	0.25 (0.17-0.36)	0.57 (0.34-0.94)	62.5	0.42 (0.23-0.78)	0.61 (0.29-1.31)
High school	14.0	0.34 (0.24-0.52)	0.70 (0.42-1.16)	77.9	0.89 (0.62-1.29)	1.15 (0.73-1.82)
Post high school	32.3	1	1	79.9	1	1
***Occupation***	***			*		
Employment	30.0	1	1	80.2	1	1
Self employment	11.4	0.30 (0.22-0.42)	0.46 (0.30-0.71)	74.8	0.73 (0.52-1.03)	0.89 (0.57-1.39)
***Parity***						
1	17.4	1	1	79.6	1	1
2	13.3	0.73 (0.52-1.02)	0.78 (0.53-1.15)	77.7	0.89 (0.63-1.27)	0.87 (0.59-1.29)
3+	13.0	0.71 (0.46-1.09)	0.93 (0.53-1.62)	66.7	0.51 (0.25-1.06)	0.65 (0.29-1.49)
***Pregnancy at risk***						
Low risk	15.9	1	1	78.3	1	1
High risk	10.2	0.60 (0.36-1.01)	0.56 (0.32-0.96)	78.3	1.00 (0.59-1.69)	1.14 (0.65-1.99)
***Household economic***	***					
Poor	9.5	0.39 (0.26-0.58)	0.73 (0.46-1.16)	74.0	0.63 (0.42-0.96)	0.69 (0.43-1.09)
Middle	11.4	0.63 (0.44-0.89)	0.88 (0.60-1.29)	78.1	0.79 (0.52-1.19)	0.80 (0.52-1.23)
Rich	21.2	1	1	81.9	1	1
***Community condition***	***		**			
Low	10.3	0.40 (0.24-0.63)	0.54 (0.32-0.92)	73.7	0.67 (0.52-1.13)	0.71 (0.47-1.07)
Middle	13.7	0.55 (0.39-0.76)	0.72 (0.50-1.04)	85.4	1.60 (0.98-2.61)	1.61 (0.97-2.67)
High	22.6	1	1	78.5	1	1
***ANC type***						
Public only	15.8	1	1	76.5	1	1
Private only	5.2	0.29 (0.12-0.69)	0.26 (0.11-0.64)	62.5	0.51 (0.26-0.99)	0.44 (0.22-0.88)
Public and private	17.4	1.12 (0.81-1.56)	0.94 (0.66-1.34)	83.7	1.57 (1.09-2.28)	1.55 (1.05-2.27)
***Total***	***15.2***			***78.3***		

*p *value for Chi square test of the null hypothesis that all true percentages are equal within a group (no tests for trends). *: *p *< 0.05; **: *p *< 0.01; ***: *p *< 0.001

In the rural area, women with overall adequate use of ANC had 5% higher household income, 23% higher cost per visit and 89% higher total cost for ANC than women with inadequate ANC use. The corresponding numbers in the urban area were 8%; 4% and 43%, respectively. The urban-rural differences between the cost ratios between adequate and inadequate user groups were increasing going from household income to cost per visit and total ANC cost. Statistically significant differences between the groups with and without adequate use was not obtained in household income, and cost per visits, but in total cost for ANC in both areas (data not shown).

## Discussion

### Rural-urban difference in adequate use of ANC

Only one study, to our knowledge, of ANC adequacy and related factors in Vietnam, that combines the number of visits, the timing of ANC visits and ANC service content and that apply theoretical models for the selection and analysis of factors has been conducted [15,16]. There, adequate ANC content was classified as "fair", "intermediate" and "poor" according to the number of services in the visits [15]. Using three indicators, total number of visits, time for first visit and specific services received during the ANC visits, this study gives a more detailed picture of ANC adequacy and its associations to selected factors in the urban and rural areas.

The estimates of overall adequate use of ANC in this study, especially in the rural area, were much lower than in other studies [11] as well as in routine administrative reports [20]. The comparatively poor use of core ANC services in the rural area was found to be the main reason for the observed large gap in overall adequate use of ANC between the two sites [21]. Better use of ANC in urban areas, as compared to rural, have been observed in many studies in low income countries [7,8,22,23]. Urban areas differ from rural areas in many respects that may contribute to differences in ANC utilization and contents. Finding associations between ANC adequacy and various factors in each area may help to understand the urban-rural disparity and also help to design interventions.

### Factors associated with ANC adequacy in the urban and the rural areas

The analyse show that most factors had odds ratios of similar direction and magnitude in the two areas, with the exception of those for pregnancy at risk and for community at middle level. However, the commonality in the statistical significance was not revealed. The statistical significance was observed for most factors in the rural area except parity and pregnancy at risk, but only for low education, low economic status and exclusive use of private ANC in the urban.

Three concepts should be discussed in relation to associations, the strength e.g. value of correlation coefficient, the direction and the statistical significance. Analyses were conducted separately in the two areas but often the same direction of the associations was observed. The urban percentages are often generally at higher levels and differences between groups are by necessity smaller and associations therefore appear less strong. There are more statistically significant differences in the rural area than in the urban. This is however, partly due to the fact that statistical significance, and *p*-values, are functions of the sample size. Since there is a substantially larger number of women in the rural group, we should expect more statistically significant results here. Similar findings have been made in other studies [24,25].

### Adequate use of ANC in relation to demographic and socioeconomic characteristics

Earlier studies have shown that women with lower education usually have less knowledge about ANC and more difficulties to get access to ANC services [16,26,27]. In this study, less educated women in both sites also had significantly lower overall adequate use of ANC. The relation between low education and low adequacy of ANC was more obvious in the rural than in the urban area. Being less educated was significantly associated with inadequate use for all three indicators of ANC in the rural area but for only two indicators in the urban, not the indicator of sufficient services. It is suggested that the less educated women should be a target group of ANC promoting program to encourage the use of sufficient services, especially in the rural areas.

Self employment was significantly related to low overall adequate use of ANC in the rural area but not in the urban. Self employed women in the rural areas attended ANC later, had fewer visits and received less sufficient ANC services than employed women. In the urban areas, self employed women had fewer visits but sufficient use of services compared to employed women. However self employment refers to different groups in the two areas, mostly farmers in the rural, and business and housework in the urban areas. Other occupational groups are small and have not been studied in detail. Important is that talking about occupation we mean different things in the two areas.

The low overall adequacy of ANC among young rural women (less than 25 years old) was mostly due to insufficient content of services. In the rural areas, the young group, 45.8% of all women, can generally be expected to have less knowledge and experiences about ANC than the older women. Ethnicity was not included in the multiple regression analyses because the group is small in the urban area and since there was no difference in overall adequate use of ANC between the majority and minority groups in the rural.

Poor women had higher risk than others not to receive overall adequate ANC in both areas. Low economic status was associated with low use of ANC visits and services. This result is similar to findings from many studies in developing countries [7]. Low economic status was however not significantly related to late first visit attendance of ANC as seen in India [27,28].

For ANC to be effective, women must receive sufficiently many of the core services during sufficiently many visits. In Vietnam, the ANC cost for a woman depends on both the number of visits used and the number of services undertaken. The ratio of total costs for ANC between the women with and without overall adequate ANC was much greater than the corresponding ratios of household income and cost per ANC visit, especially in the rural area. For poor women, financial constraints might not be important for a single ANC visit but for repeated visits and services [16]. In the rural area, the risk of low overall adequate ANC was higher not only among women at low economic condition but also for women at middle level. The results also suggest that the cost may be a barrier against optimal use of ANC for economically disadvantaged groups. Financial support might help them to have more adequate use of ANC.

The association of community economic condition with overall adequate use of ANC in the two areas was not as obvious as the association at household level. The community condition was significantly but weakly related only to some indicators of ANC adequacy in the rural areas. One reason might be that distance to the nearest health facilities, itself related to ANC utilization, is more variable between the three community groups in the rural area.

### Adequate use of ANC in relation to obstetric and health seeking factors

The risk for complications in a second pregnancy is usually much lower if the first pregnancy and birth was uncomplicated than if it was not [19]. Multiparous women have earlier reported fewer ANC visits than nulliparous possibly also because they had experienced previous pregnancies [27,29]. Here, a significant association between high parity and insufficient use of core services was found among the urban women who had more than two childbirths. There was no statistical evidence for late first visit attendance of ANC among multiparous women as found in previous studies in Vietnam [16,29].

The time for first visit ANC during the first trimester was significantly related to the use of many ANC visits and services, especially in the rural area. This is natural since women who attended ANC within the first trimester, have more time for further visits and opportunity to receive more services than those who started ANC later. Educating and encouraging women with low education and self employed women to attend ANC early are necessary to improve ANC adequacy in rural areas.

It is noticeable that women with high risk pregnancy did not report significantly more visits and services than the low risk women in either area. In the rural area, they even had less overall adequate ANC than women with low risk pregnancy. The explanation is likely to be that the "high risk pregnancy" concept is not known by the women and ANC providers. There was only a very low percentage of women, especially in the rural areas, who received prenatal health education during ANC visits [21]. As a consequence, the women at high risk were not aware of their situation and need for strict adherence to ANC recommendations.

A low percentage of rural women used all core ANC services at least once during pregnancy. The number of women who received all core services in every visit must be even lower. Simple services, such as blood pressure measurement and urine test assessment have been considered as cost-effective antenatal services as they detect early severe pregnancy complications like pre-eclampsia and eclampsia. The use of these services once during pregnancy is not enough and a number of high risk rural women are likely not to be identified.

Similar to findings in a study in Brazil [30], the women who exclusively used ANC in the private sector had higher risk of receiving low overall adequate ANC both in rural and urban areas. The exclusive use of private ANC was not significantly associated with late first visit attendance or less use of visits but to insufficient use of services, suggesting poor service content of care and a low compliance to national guidelines in the private sector, possibly due to weak regulation and control [31]. The large share of private ANC use among rural women might partly explain the low overall adequacy of ANC in this area. There is a need to improve the adherence of private health workers to ANC guidelines, especially in rural areas.

The use of private health care is widespread in rural Vietnam, not because it is cheaper or offers better services, but possibly due to the convenience and the availability [31]. Many pregnant women sometimes just want to have an ultrasound scan done which is available in very few commune health centers. They can get it easier in private health facilities. It is interesting that in both sites, the women with low education used ANC only from the public sector more often than the others while the highly educated women used private ANCs more frequently (*p *< 0,01; results not shown). It seems that the women use ANC at private health care facilities because they pay more attention to single specific services such as ultrasound examination than to the overall adequacy. The effect can be that the more private ANC women use, the less adequate ANC they receive.

In the rural areas, women used more private ANCs although the average cost for ANC utilization was 1.5 times that in the public sector [21]. This creates a vicious spiral: rural women, with lower incomes, use more private services with higher costs and lower adequacy. As a result, the rural women with limited resources, receive fewer visits and services at private facilities than they could have received in the public sector. In order to improve ANC adequacy in the rural areas, the quality of care, private as well as public, should be improved in parallel with the encouragement to use of ANC at public primary health care facilities. The lower overall adequate use of ANC in disadvantaged groups in the rural areas should be seriously considered in policy making and planning. It is necessary to organize media campaigns focusing on these groups about the importance of early attendance, timing and the basic antenatal care service contents during pregnancy. Antenatal care provision must be available on all weekdays in CHCs and the form for financial support for antenatal care of poor and near poor women should be seriously considered. Pregnancy health insurance or fee exemption could be used.

### Strength and limitation

The study was conducted within the framework of two HDSS with quite large sample sizes. This means that the context is known and information about demography as well as social and economic characteristics of households is available. Data was collected by interviewers who have been trained carefully and had substantial experiences in household interviews. A question about the timing of ANC was asked only for the first visit and the information about services at the individual visits is not available. Only overall service use during the pregnancy, yes or no, was recorded. Recall bias is likely to be small with the three-month cycle compared to other studies where all questions have been asked after delivery. ANC adequacy was assessed using several indicators unlike earlier study that only used the number of ANC visits.

The study was carried out in two specific areas that can be considered to be in a better situation with respect to socio-economy than other parts of Vietnam. Broad generalizations outside the investigated areas are hardly useful for local policy and strategy and intervention. However, it might be possible to claim applicability of the results to other specified areas based on criteria for contextual similarity e.g. regarding demography, economy, health care structure etc.

## Conclusions

The rural and urban settings show different levels of overall ANC adequacy and different associations to its determinants. Women with low education, low economic status or exclusive use of private ANC have higher risk of receiving inadequate ANC in both urban and rural areas. For rural women the study supports that getting pregnant before 25 years old, self employment or living in poor communities also had lower chance to receive overall adequate ANC.

To improve the quality and utilization of the ANC program, young, poor and less educated women in rural areas should be prioritized focusing on encouraging early attendance of ANC and use of core services. Financial support for women economically disadvantaged groups to use adequate ANC should be considered. Providers of ANC should be educated and otherwise influenced to provide sufficient core services. Better control is needed to strengthen adherence to ANC content guidelines particularly in the private sector.

## Competing interests

The authors declare that they have no competing interests.

## Authors' contributions

TKT carried out the study and participated in data analysis and interpretation, drafted and revised the manuscript. MP was responsible for supervision of the study, participated in the conception and design of the research, analyses and interpretation of the data and revising the manuscript. KG and HDN contributed to study design, coordination of the study and to revision of the manuscript. HA involved in interpretation of the data, drafting and revising the manuscript. All authors read and approved the final manuscript.

## Pre-publication history

The pre-publication history for this paper can be accessed here:

http://www.biomedcentral.com/1472-6963/12/40/prepub

## References

[B1] The State of the world's children Maternal and Newborns health, 2008 2009 New York: Division of Communication, UNICEF

[B2] Zanconato G Antenatal care in developing countries: the need for a tailored model Semin Fetal Neonatal Med 2006 11 1 15 20 10.1016/j.siny.2005.10.002 16364704

[B3] WHO, UNICEF Antenatal Care in developing Countries: Promises, Achievements and Missed Opportunities: An Analysis of Trends, Levels, and Differentials: 1990-2001 2003 Geneva, New York: WHO & UNICEF

[B4] Navaneetham K Dharmalingam A Utilization of maternal health care services in Southern India Soc Sci Med 2002 55 10 1849 1869 10.1016/S0277-9536(01)00313-6 12383469

[B5] Paredes I Factors associated with inadequate prenatal care in Ecuadorian women Int J Gynaecol Obstet 2005 88 2 168 172 10.1016/j.ijgo.2004.09.024 15694103

[B6] Alam AY Comparative study of knowledge, attitude and practices among antenatal care facilities utilizing and non-utilizing women J Pak Med Assoc 2005 55 2 53 56 15813628

[B7] Simkhada B Factors affecting the utilization of antenatal care in developing countries: systematic review of the literature J Adv Nurs 2008 61 3 244 260 10.1111/j.1365-2648.2007.04532.x 18197860

[B8] Kishk NA Knowledge, attitudes and practices of women towards antenatal care: rural-urban comparison J Egypt Public Health Assoc 2002 77 5-6 479 498 17216974

[B9] Alexandre PK Prenatal care utilization in rural areas and urban areas of Haiti Rev Panam Salud Publica 2005 18 2 84 92 16156958 10.1590/s1020-49892005000700002

[B10] Ministry of Health (MoH) National Guideline on reproductive health services 2003 Hanoi: Medical Publishing House 45 56

[B11] Vietnam Ministry of Health (MoH) and General Statistics Office (GSO) Results of the National Health Survey 2001-2002 2003 Hanoi: Medical Publishing House

[B12] Ministry of Health (MoH) Heath Statistical Profile 2001-2005 2006 Hanoi: MoH

[B13] Swenson IE Factors related to the utilization of prenatal care in Vietnam J Trop Med Hyg 1993 96 2 76 85 8096252

[B14] Toan NV Utilization of reproductive health services in a mountainous area in Vietnam Southeast Asian J Trop Med Public Health 1996 27 2 325 332 9279997

[B15] Trinh LT Michael John D Byles J Antenatal care adequacy in three provinces of Vietnam: Long An, Ben Tre, and Quang Nga Public Health Rep 2006 121 4 468 475 16827450 10.1177/003335490612100417 PMC1525361

[B16] Trinh LT Dibley MJ Byles J Determinants of antenatal care utilization in three rural areas of Vietnam Public Health Nurs 2007 24 4 300 310 10.1111/j.1525-1446.2007.00638.x 17553019

[B17] Chuc NT Diwan V FilaBavi, a demographic surveillance site, an epidemiological field laboratory in Vietnam Scand J Public Health Suppl 2003 62 3 7 14578073 10.1080/14034950310015031

[B18] Tran TK Preliminary Results from the baseline survey of DodaLab HDSS in 2007 2008 Hanoi Medical University: Hanoi

[B19] Dangal G High-Risk Pregnancy The Internet Journal of Gynecology and Obstetrics 2007 7 1

[B20] Ministry of Health (MoH) The Health Statistics Year Book 2009 2011 Hanoi

[B21] Tran TK Urban - rural disparities in antenatal care utilization: a study of two cohorts of pregnant women in Vietnam BMC Health Serv Res 2011 11 1 120 10.1186/1472-6963-11-120 21605446 PMC3224373

[B22] Collin SM Anwar I Ronsmans C A decade of inequality in maternity care: antenatal care, professional attendance at delivery, and caesarean section in Bangladesh (1991-2004) Int J Equity Health 2007 6 9 10.1186/1475-9276-6-9 17760962 PMC2014749

[B23] Sepehri A How important are individual, household and commune characteristics in explaining utilization of maternal health services in Vietnam? Social Science & Medicine 2008 67 2008 1009 1017 18635302 10.1016/j.socscimed.2008.06.005

[B24] Nisar N White F Factors affecting utilization of antenatal care among reproductive age group women (15-49 years) in an urban squatter settlement of Karachi J Pak Med Assoc 2003 53 2 47 53 12705483

[B25] Celik Y Hotchkiss DR The socio-economic determinants of maternal health care utilization in Turkey Soc Sci Med 2000 50 12 1797 1806 10.1016/S0277-9536(99)00418-9 10798333

[B26] Titaley CR Dibley MJ Roberts CL Factors associated with underutilization of antenatal care services in Indonesia: results of Indonesia Demographic and Health Survey 2002/2003 and 2007 BMC Public Health 2010 10 485 10.1186/1471-2458-10-485 20712866 PMC2933719

[B27] Shariff A Singh G Determinants of Maternal Health Care Utilization in India: Evidence from a Recent Household Survey 2002 National Council of Applied Economic Research: New Dehli S. no.85, Editor

[B28] Pallikadavath S Foss M Stones RW Antenatal care: provision and inequality in rural north India Soc Sci Med 2004 59 6 1147 1158 10.1016/j.socscimed.2003.11.045 15210087

[B29] Graner S Maternal health care professionals' perspectives on the provision and use of antenatal and delivery care: a qualitative descriptive study in rural Vietnam BMC Public Health 2010 10 608 10.1186/1471-2458-10-608 20946681 PMC3091560

[B30] Ribeiro ER Risk factors for inadequate prenatal care use in the metropolitan area of Aracaju Northeast Brazil. BMC Pregnancy Childbirth 2009 9 31 10.1186/1471-2393-9-31 PMC2720914 19622174

[B31] Tuan T Comparative quality of private and public health services in rural Vietnam Health Policy Plan 2005 20 5 319 327 10.1093/heapol/czi037 16076933

